# Efficacy and Safety of a Combination of Shenmai Injection plus Chemotherapy for the Treatment of Lung Cancer: A Meta-Analysis

**DOI:** 10.1155/2021/7929165

**Published:** 2021-04-13

**Authors:** Guo-wei Qin, Tong-tong Xu, Xiang-wei Lv, Shi-min Jiang, Ke-jia Zhang, Ming Xu, Lu Fu, Qi Wu, Yao Zhou

**Affiliations:** ^1^Science and Technology Department of Guilin Medical University, Guilin 541001, Guangxi Zhuang Autonomous Region, China; ^2^First Affiliated Hospital of Guilin Medical University, Guilin 541001, Guangxi Zhuang Autonomous Region, China; ^3^Department of Pathophysiology, Xuzhou Medical University, Xuzhou 221009, China; ^4^Laboratory of Clinical and Experimental Pathology, Xuzhou Medical University, Xuzhou 221009, China; ^5^Department of Physiology, Xuzhou Medical University, Xuzhou 221009, China

## Abstract

**Objective:**

To perform a systematic evaluation of the efficacy and safety of combined treatment of Shenmai injection and chemotherapy for lung cancer.

**Methods:**

A literature search for randomized controlled trials (RCTs) describing the treatment of lung cancer by Shenmai injection and chemotherapy or chemotherapy alone was performed using the PubMed, Cochrane Library, China National Knowledge Infrastructure (CNKI), Value In Paper (VIP), China BioMed, and Wanfang databases. The databases were searched for entries published before September 1, 2019.

**Results:**

Thirty-seven RCTs, comprising a total of 2808 cases, were included in the present meta-analysis. Of these, 1428 cases were treated by Shenmai injection plus chemotherapy, and 1380 cases were treated only by chemotherapy. The results of meta-analysis showed that the combined treatment (Shenmai injection plus chemotherapy) increased the short-term efficacy of treatment (relative risk [RR] = 1.183, 95% confidence interval [CI] = 1.043–1.343, *P* < 0.01) and improved patients' quality of life (RR = 1.514, 95%CI = 1.211–1.891, *P* < 0.01) compared with chemotherapy alone. With regard to the adverse effects, the combined treatment markedly reduced the incidence of white blood cell (WBC) reduction (RR = 0.846, 95%CI = 0.760–0.941, *P* < 0.01), platelet reduction (RR = 0.462, 95% CI = 0.330–0.649, *P* < 0.01), and hemoglobin reduction (RR = 0.462, 95% CI = 0.330–0.649, *P* < 0.01) and alleviated drug-induced liver injury (RR = 0.677, 95%CI = 0.463–0.990, *P* < 0.05). However, it did not offer a significant protective effect (RR = 0.725, 95%CI = 0.358–1.468, *P* < 0.05). The effect of the combined treatment on the occurrence of vomiting was considerable (RR = 0.889, 95%CI = 0.794–0.996, *P* < 0.05), and the combined treatment markedly increased the immunity of patients with lung cancer.

**Conclusion:**

The combined treatment of Shenmai injection plus chemotherapy enhanced the short-term efficacy of chemotherapy, improved the patient quality of life, alleviated the adverse effects of chemotherapeutics, and increased the patient immunity. These results should be confirmed by large-scale, high-quality RCTs.

## 1. Introduction

Lung cancer is a malignant disease with the highest death rate and the second highest incidence worldwide [1]. There are two types of lung cancer: small-cell lung cancer and non-small-cell lung cancer (NSCLC); NSCLC accounts for 85% of all lung cancers and has a death rate as high as 80%–90% [2]. Small-cell lung cancers are sensitive to chemotherapy; therefore, they are treated mainly by chemotherapy. Early stage lung cancers can still be treated surgically. However, approximately 70% of patients with a lung cancer are in an advanced stage upon diagnosis and are treated mainly by chemotherapy [3, 4]. Although chemotherapy has a significant effect on the reduction of the number of tumors, severe adverse effects always occur, including reductions in neutrophil granulocytes and platelets and unendurable anemia, emesis, and nausea [5]. Consequently, patients are forced to query their treatment, and the final efficacy is affected. Therefore, one aim of research is to find drugs that enhance efficacy of chemotherapeutics and alleviate their toxicity.

Shenmai injection is a Chinese medicine preparation; its constituent bioactive ingredients are panaxan, ginsenoside, ophiopogonin, ophiopogonanone, and Ophiopogon polysaccharides [6]. Shenmai injection was reported to activate the body's immune function and improve internal microcirculation and has been used widely as a clinical treatment for coronary artery disease [7, 8]. Further in-depth studies showed that Shenmai injection could inhibit infiltration and metastasis, promote apoptosis in tumor cells, and enhance the efficacy of chemotherapeutics while alleviating their toxicity [9, 10]. Therefore, in the treatment of lung cancer, Shenmai injection can be used as an adjuvant medicine to alleviate the adverse effects of chemotherapeutics and improve patients' quality of life. In recent years, although there have been several RCTs investigating the combination treatment of lung cancer with Shenmai injection plus chemotherapy, most of these trials have a small sample size and the evidence has not been convincing. In the present study, RCTs analyzing the combination treatment of lung cancer with Shenmai injection plus chemotherapeutics were identified and evaluated for their efficacy and safety using the Cochrane assessment method. The results of this study will provide evidence to support the clinical application of combined treatment.

## 2. Data and Methods

### 2.1. Inclusion Criteria

#### 2.1.1. Type of Research

RCTs describing combination treatments of Shenmai injection and chemotherapeutics published before September 1, 2019, were searched for, irrespective of the type of document and or the language of publication.

#### 2.1.2. Study Subjects

Patients with a definitive diagnosis of lung cancer based on the lung cancer diagnosis criteria.

#### 2.1.3. Preventative Measures

The experimental group was treated with Shenmai injection plus chemotherapy. The control group was treated with chemotherapy alone.

#### 2.1.4. Exclusion Criteria

1. Indefinite diagnosis
2. The use of other therapies, including Chinese medicine and acupuncture
3. No treatment with chemotherapeutics
4. Animal experiments
5. Full-length paper was unavailable; duplicate results; or results with missing data

#### 2.1.5. Outcome Indicators

1. Short-term efficacy: determined by the objective evaluation standard for solid tumors prepared by EFCIST or the response criteria in solid tumors prepared by WHO [11]. Efficacy = complete remission (CR) + partial remission (PR).
2. Kamofsky Performance Status (KPS score) (including stabilization and improvement): an increase in the KPS by more than 10 points after treatment compared with the score before treatment was considered an improvement, whereas a reduction in KPS by more than 10 points indicated degradation; and an increase or reduction in KPS of less than 10 points before and after treatment suggested stabilization.
3. Immune function: including assessment of T-lymphocytes, CD3^+^, CD4^+^, and CD8^+^.
4. Adverse events: including assessment of changes in white blood cells (WBCs), hemoglobin, platelets, renal function, liver function, and incidence of nausea and emesis.

#### 2.1.6. Data Source and Search Strategy

This search was conducted in accordance with the Preferred Reporting Items for Systematic Reviews and Meta-Analyses (PRISMA) statement for the conduct of meta-analyses of intervention studies. Literature published before September 1, 2019, describing chemotherapy and Shenmai injection was searched for in the China National Knowledge Infrastructure (CNKI), Value In Paper (VIP), WanFang, CBM, PubMed, and Cochrane Library databases. The search was performed using the following key words and free text: lung neoplasm OR pulmonary neoplasm OR thoracic neoplasm OR pulmonary cancer OR lung cancer OR thoracic cancer OR lung carcinoma OR pulmonary carcinoma OR thoracic carcinoma AND (Shenmai injection OR Shenmai).

#### 2.1.7. Quality Assessment of Methodology and Data Extraction of Studies Included in the Meta-analysis

Methodological quality was evaluated using the criteria in the Cochrane Handbook version 5.1. The indicators under evaluation included: (1) method of randomization assignment; (2) allocation concealment; (3) whether blinded methods were adopted; (4) whether research results were reported selectively; (5) whether study results were complete; (6) whether there was any possibility of deviation.

#### 2.1.8. Data Extraction

Data were assessed collaboratively by two independent research fellows. The two research fellows extracted data in the relevant literature and compiled a thorough summary and performed a cross check. Any inconsistencies were resolved by resolution with a third party. The data to be extracted were as follows:

1. Basic information on the literature: title, author, date of publication, volume, issue, and page number.
2. Characteristics of research: specific method for study procedures, including randomization control, blinding, and follow-up visits.
3. Basic information on study subjects: sample size, age, sex, criteria for diagnosis and exclusion, and course of treatment.
4. Preventative measures: medication status of the combined treatment with chemotherapy plus Shenmai injection and chemotherapy alone, including drug name, dosage, components, frequency of application, and course of treatment.
5. Indicators of observation: efficacy assessment, KPS score, immune function, and adverse events.

#### 2.1.9. Statistical Analysis

Revman version 5.3 and Stata version 12.0 were used to analyze the data. Numerical data were analyzed by relative risk (RR). Metering data were analyzed by the standardized mean difference (SMD). The significance level was set as *α* = 0.05 and the confidence interval (CI) considered for both RR and SMD was 95%. Heterogeneity was tested by the chi-square and *I*^2^ tests. Values of *P* > 0.1 and I^2^ ≤ 50%, respectively, suggested low heterogeneity among the studies and the fixed effect model was chosen for the statistical analysis. Values of *P* > 0.1 and *I*^2^>50% indicated large heterogeneity among the studies, and the random effects model was chosen for the statistical analysis. Publication bias was evaluated by the Begg test, with a value of *P* < 0.05 considered to represent a significant difference. When sufficient studies were included, a sensitivity test was used to examine the stability of the results.

## 3. Results

### 3.1. Results of the Literature Search

**Figure 1:**
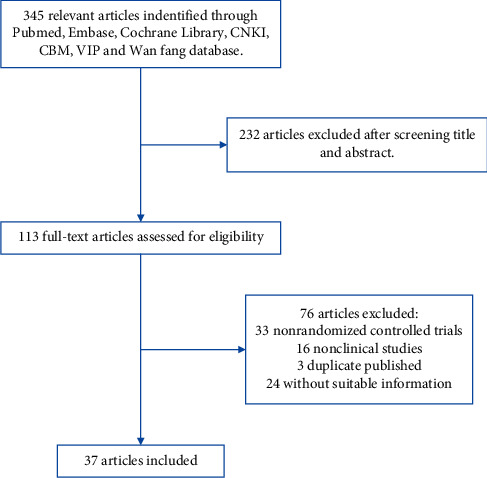
Literature retrieval and screening process.

In total, our search strategy returned 345 publications, of which 232 were excluded after filtering the titles and abstracts. The full text of the remaining 113 publications was read, and 33 publications describing nonrandomized control trials, 16 publications describing animal experiments, 3 duplicate publications, and 24 publications with ambiguous preventative measures or diagnosis were excluded. Finally, 37 studies were included in the present meta-analysis (Figure 1).

### 3.2. Basic Characteristics of Studies Included in the Present Meta-analysis

The basic information included the authors, date of publication, sample size, clinical stage, therapeutic strategy, and drug dosage. In the 37 studies included in the meta-analysis, the observation group contained 1428 cases and the control group contained 1380 cases. The chemotherapy regimens included GP (gemcitabine + cis-platinum), NP (navelbine + cis-platinum), TP (taxol + carboplatin), DP (docetaxel + cis-platinum), and TC (taxol + cis-platinum). Shenmai injection was applied intravenously at a dosage of 40 mL/day–100 mL/day. The duration of treatment was between 7 and 28 days, inclusive (Table 1).

### 3.3. Quality Assessment of Literature Included

**Figure 2:**
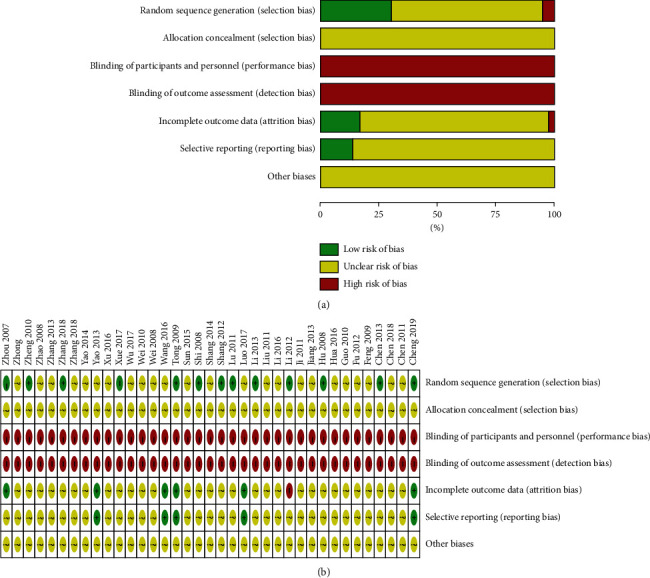
Bias risk analysis of included studies. (a) Methodological quality assessment of all included studies. (b) Methodological quality summary of each included study. +: L (low risk of bias); ?: U (unclear risk of bias); −: H (high risk of bias).

The quality of literature included was analyzed in accordance with the criteria for RCTs in The Cochrane Handbook for Systematic Reviews of Interventions (version 5.1.0). Among the 37 RCTs included, 35 mentioned the word random and 7 mentioned the specific method of randomization. None of these 37 RCTs mentioned allocation concealment. All included RCTs did not adapt blind methods. In addition, owing to data insufficiency, we could not determine whether there was any form of bias. The publication bias of the literature included was evaluated; the results are shown in Figure 2(a) and Figure 2(b).

### 3.4. Short-Term Efficacy

**Figure 3:**
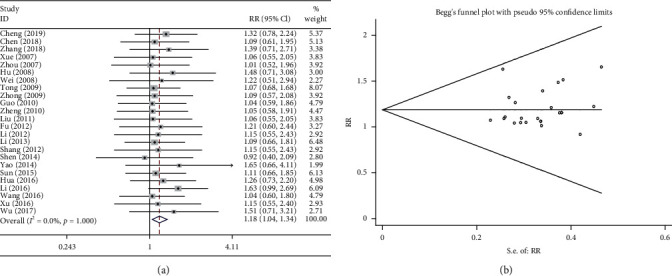
Meta-analysis of short-term efficacy in the included studies. (a) Forest plots of the comparisons of the short-term efficacy between the Shenmai injection plus chemotherapy group and the chemotherapy alone group. (b) Funnel plots assessing the publication bias using Begg's rank correlation test.

Twenty-four studies compared the short-term efficacy of the combination treatment (Shenmai injection plus chemotherapy) and of chemotherapy alone. The results of the heterogeneity test were *P* > 0.1 and *I*^2^ = 0%, suggesting that there was low heterogeneity; therefore, a fixed effects model was adopted. The pooled results showed that first-line treatment with the combination treatment of Shenmai injection plus platinum-containing chemotherapy had significantly higher short-term efficacy than chemotherapy alone (RR = 1.183, 95%CI = 1.043–1.343, *P* < 0.01, Figure 3(a)). The results of the Begg test did not suggest the existence publication bias (*P* > 0.1, Figure 3(b)).

### 3.5. KPS Score

**Figure 4:**
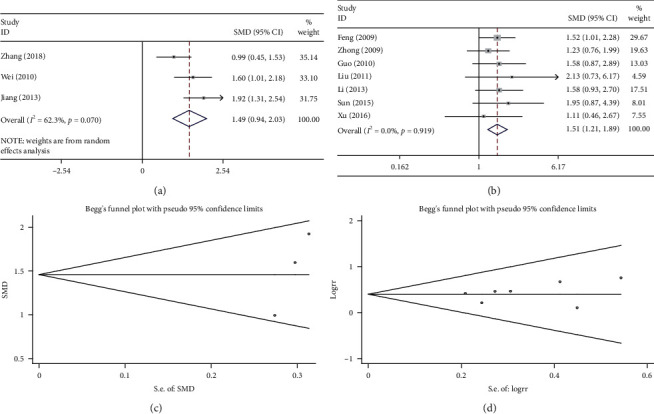
Meta-analysis of KPS score in the included studies. (a) and (c) Forest plots of the comparisons of the KPS score between the Shenmai injection plus chemotherapy group and the chemotherapy group. (b) and (d) Funnel plots assessing publication bias using the Begg's rank correlation test.

Three studies compared the KPS score of the combination treatment (Shenmai injection plus chemotherapy) and chemotherapy alone. The results of heterogeneity test were *P* < 0.1 and *I*^2^ = 62.3%; therefore, a random effects model was used. The pooled results showed that the combination treatment significantly increased patients' KPS score compared with chemotherapy alone (SMD = 1.487, 95%CI = 0.943–2.030, *P* < 0.01, Figure 4(a)). As shown in Figure 4(c), the results of Begg test did not reveal any publication bias (*P* > 0.1).

Seven studies calculated the KPS score and compared the efficacy of the combination treatments. The improvement in quality of life for the combination treatment and chemotherapy alone was evaluated. The results of the heterogeneity test were *P* > 0.1 and *I*^2^ = 0%; therefore, a fixed effects model was used. The pooled results showed that the combination treatment significantly enhanced patient quality of life compared with chemotherapy alone (RR = 1.514, 95%CI = 1.211–1.891, *P* < 0.01; Figure 4(b)). As shown in Figure 4(d), the results of Begg test did not reveal any publication bias (*P* > 0.1).

### 3.6. Toxic and Side Effects

#### 3.6.1. Reduction in White Blood Cell Count

**Figure 5:**
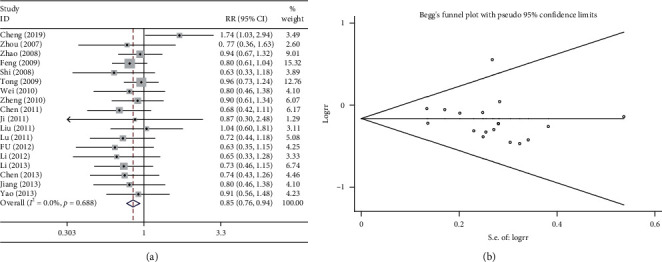
Meta-analysis of the reduction in WBCs. (a) Forest plots revealing the comparisons of the reduction in WBCs between the Shenmai injection plus chemotherapy group and the chemotherapy alone group. (b) Funnel plots assessing publication bias.

A reduction in WBCs is a commonly observed toxic adverse effect of chemotherapy. In 18 of the 37 RCTs, the reduction in WBCs following the combination treatment (Shenmai injection plus chemotherapy) was compared with that of chemotherapy alone. The results of the heterogeneity test were *P* > 0.1 and *I*^2^ = 0%; therefore, a fixed effects model was used. The pooled results showed that the combination treatment significantly reduced the occurrence of WBC reduction (RR = 0.846, 95%CI = 0.760–0.941, *P* < 0.01, Figure 5(a)) compared with chemotherapy alone. As shown in Figure 5(b), the results of Begg test did not reveal any publication bias (*P* > 0.1).

#### 3.6.2. Reduction in Hemoglobin

**Figure 6:**
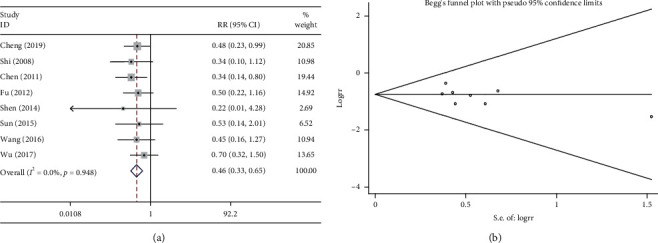
Meta-analysis of the reduction in hemoglobin. (a) Forest plots of the comparisons of the reduction in hemoglobin between the Shenmai injection plus chemotherapy group and the chemotherapy alone group. (b) Funnel plots assessing publication bias.

Eight studies compared the reduction in hemoglobin following the combination treatment (Shenmai injection plus chemotherapy) and following chemotherapy alone. The heterogeneity test suggested very low heterogeneity (*P* > 0.1 and *I*^2^ = 0%); therefore, a fixed effects model was used. The pooled results showed that the combination treatment significantly reduced the incidence of hemoglobin reduction compared with chemotherapy alone (RR = 0.462, 95%CI = 0.330–0.649, *P* < 0.01, Figure 6(a)). As shown in Figure 6(b), the results of Begg test did not reveal any publication bias (*P* > 0.1).

#### 3.6.3. Reduction in Platelets

**Figure 7:**
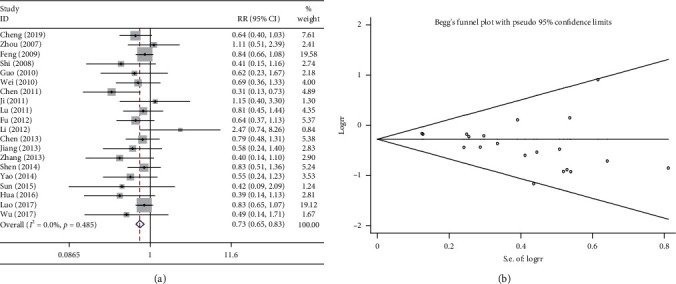
Meta-analysis of platelet reduction. (a) Forest plots of the comparisons of platelet reduction between the Shenmai injection plus chemotherapy group and the chemotherapy alone group. (b) Funnel plots assessing publication bias.

Twenty studies compared the reduction in platelet following the combination treatment (Shenmai injection plus chemotherapy) and chemotherapy alone. The heterogeneity test suggested very low heterogeneity (*P* > 0.1, *I*^2^ = 0%); therefore, a fixed effects model was used. The pooled results showed that the combination treatment significantly decreased the reduction in platelet levels compared with chemotherapy alone (R = 0.462, 95%CI = 0.330–0.649, *P* < 0.01, Figure 7(a)). As shown in Figure 7(b), the results of Begg test did not reveal any publication bias (*P* > 0.1).

#### 3.6.4. Liver Function

**Figure 8:**
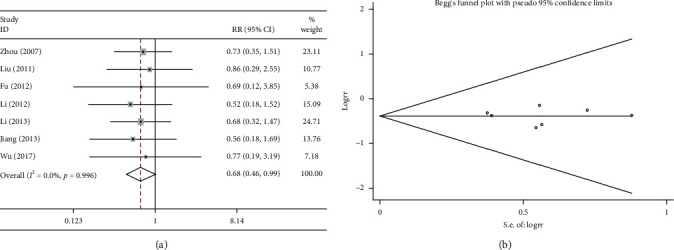
Meta-analysis of the reduction in liver function. (a) Forest plots revealing the comparisons of the reduction in liver function between the Shenmai injection plus chemotherapy group and the chemotherapy alone group. (b) Funnel plots assessing publication bias.

Drug-induced liver injury is a frequently observed phenomenon in patients with lung cancer. Seven studies in the analysis compared liver function injury following the combination treatment (Shenmai injection plus chemotherapy) and following chemotherapy alone. The heterogeneity test suggested very low heterogeneity (*P* > 0.1 and *I*^2^ = 0%); therefore, a fixed effects model was used. Liver injury in the patients with lung cancer receiving the combination treatment was significantly reduced compared with that in patients receiving chemotherapy alone (RR = 0.677, 95%CI = 0.463–0.990, *P* < 0.05; Figure 8(a)). As shown in Figure 8(b), the results of Begg test did not reveal any publication bias (*P* > 0.1).

#### 3.6.5. Renal Function

**Figure 9:**
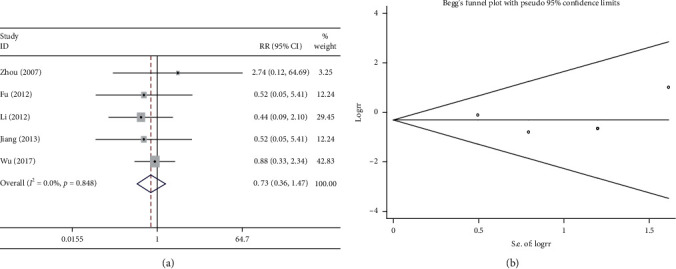
Meta-analysis of the reduction in renal function. (a) Forest plots of comparisons of the reduction in renal function between the Shenmai injection plus chemotherapy group and the chemotherapy group. (b) Funnel plots assessing publication bias.

Five studies compared renal function injury following the combination treatment (Shenmai injection plus chemotherapy) with that following chemotherapy alone. The heterogeneity test suggested very low heterogeneity (*P* > 0.1 and *I*^2^ = 0%); therefore, a fixed effects model was used. The combination treatment did not reduce the incidence of renal function injury compared with chemotherapy alone (RR = 0.725, 95%CI = 0.358–1.468, *P* < 0.05, Figure 9(a). As shown in Figure 9(b), the results of the Begg test did not reveal any publication bias (*P* > 0.1).

#### 3.6.6. Emesis

**Figure 10:**
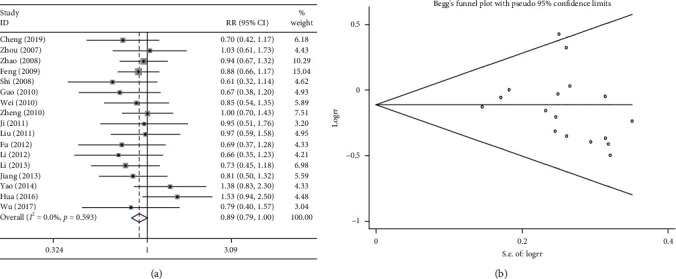
Meta-analysis of vomiting as a response to treatment. (a) Forest plots of the comparisons of the reduction in vomiting between the Shenmai injection plus chemotherapy group and the chemotherapy alone group. (b) Funnel plots assessing publication bias.

Seventeen studies compared vomiting in patients receiving the combination treatment (Shenmai injection plus chemotherapy) and in patients receiving chemotherapy alone. The results of heterogeneity test did not suggest any heterogeneity (*P* > 0.1; I^2^ = 0%); therefore, a fixed effects model was used to merge the data. The pooled results showed that the number of cases in which vomiting was a response to treatment in patients receiving the combination treatment was much lower than in patients receiving chemotherapy alone (RR = 0.889, 95%CI = 0.794–0.996, *P* < 0.05; Figure 10(a)). As shown in Figure 10(b), the results of Begg test did not reveal any publication bias (*P* > 0.1).

### 3.7. Immune Function

#### 3.7.1. CD3^+^

**Figure 11:**
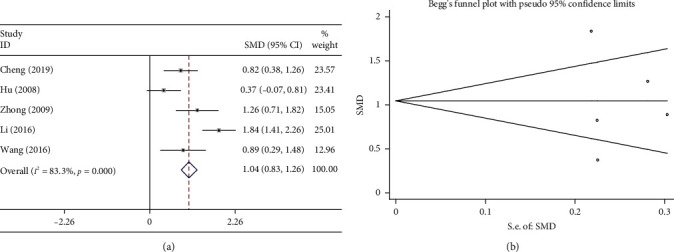
Meta-analysis of CD^3+^. (a) Forest plots of the comparisons of CD^3+^ between the Shenmai injection plus chemotherapy group and the chemotherapy group. (b) Funnel plots assessing publication bias.

The ratio of CD^3+^ to CD^4+^ is used as a clinical assessment of the immune function of patients with lung cancer. Five studies documented the changes in CD^3+^ in patients receiving the combination treatment (Shenmai injection plus chemotherapy) and in patients receiving chemotherapy alone. The results of heterogeneity test suggested large heterogeneity (*P* < 0.1; *I*^2^ = 83.3%); therefore, a random effects model was used to analyze the data. The results of the meta-analysis showed that the combination treatment markedly increased the proportion of CD^3+^ compared with chemotherapy alone (SMD = 1.044, 95%CI = 0.830–1.258, *P* < 0.05, Figure 11(a)). As shown in Figure 11(b), the results of Begg test did not reveal any publication bias (*P* > 0.1).

#### 3.7.2. CD^4+^

**Figure 12:**
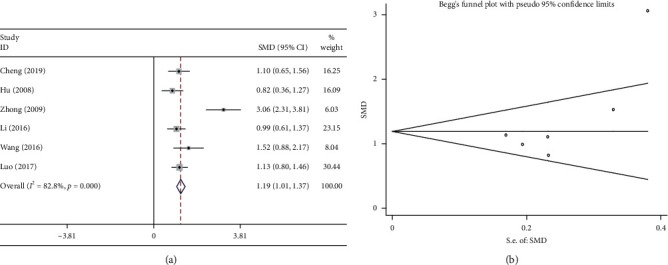
Meta-analysis of CD^4+^. (a) Forest plots of the comparisons of CD^4+^ between the Shenmai injection plus chemotherapy group and the chemotherapy alone group. (b) Funnel plots assessing publication bias.

Six studies compared the proportion of CD^4+^ in patients receiving the combination treatment (Shenmai injection plus chemotherapy) and in patients receiving chemotherapy alone. The results of the heterogeneity test suggested large heterogeneity (*P* < 0.1; *I*^2^ = 82.8%); therefore, a random effects model was used to analyze the data. The results of the meta-analysis showed that the proportion of CD4^+^ in patients receiving the combination treatment was significantly higher than that in patients receiving chemotherapy alone (SMD = 1.190, 95%CI = 1.007–1.373, *P* < 0.05; Figure 12(a)). As shown in Figure 12(b), the results of Begg test did not reveal any publication bias (*P* > 0.1).

## 4. Discussion

Recent in-depth studies of the use of Chinese medicine for the treatment of lung cancer have elucidated its clinical effects; as such, it has emerged as a research hotspot [49]. Chinese medicine can alleviate the toxic adverse effects associated with the current clinical treatments, extend the survival time of patients with cancer, increase patient quality of life, and enhance immune function; thus, it may compensate for the shortage of available treatments for lung cancer in Western medicine and is emerging as an indispensable component of cancer treatment [50].

Modern pharmacological studies have shown that Shenmai injection can modulate immune function, enhance the body's immunity, promote the activity of natural killer (NK) cells, alleviate the toxic and adverse effects of chemotherapy, increase the patient's tolerance to chemotherapeutics, and exert anti-inflammatory and antitumor effects [9]. Clinical trial results also showed that Shenmai injection could act synergistically with chemotherapeutics in lung cancer treatment. Although this treatment was characterized by lower side effects and higher patient tolerance, there is a lack of high-quality supporting evidence.

The present meta-analysis included a total of 37 studies; all investigated either combination treatments of Shenmai injection plus chemotherapy and chemotherapy alone (as a control). For an analysis of the short-term efficacy, we pooled the data from the documented effective and ineffective cases together. The effective and ineffective cases were determined by the size of tumors, which is a relatively visual indicator. The results of the present meta-analysis showed that the combination treatments (Shenmai injection plus chemotherapy) could enhance the short-term efficacy compared with that of chemotherapy alone and suggested that the efficacy of the combined treatments was very good.

We adopted the internationally renowned KPS score as an evaluation of quality of life. The pooled results showed that the KPS score of patients with lung cancer receiving the combination treatment was markedly increased compared with that of patients receiving chemotherapy. This suggested that the treatment of lung cancer with Shenmai injection plus chemotherapy had beneficial effects on the patients' quality of life.

During chemotherapy in patients with lung cancer, marrow inhibition, such as reductions in WBCs, hemoglobin, and platelets, occurs frequently. Other adverse effects, including renal function and liver function impairment, nausea, and emesis, are also frequently observed. The results of the present meta-analysis showed that the use of a combination treatment (Shenmai injection plus chemotherapy) for patients with lung cancer could significantly decrease the reductions in WBCs, hemoglobin, and platelets. Although this treatment strategy could protect liver function and markedly reduce the incidence of nausea and emesis, its effects on the protection of renal function were insignificant. The results suggested that Shenmai injection allowed good control of side effects in patients with lung cancer receiving chemotherapy.

Patients with lung cancer are usually kept in a state of immunosuppression. Immune malfunction is an important cause of the persistent proliferation and invasion of tumor cells. T-lymphocytes play a key role in the immunological surveillance of malignant tumors. Immune cell malfunction of patients with lung cancer may reduce the CD^3+^ to CD^4+^ ratio. The results of the meta-analysis showed that the combination treatment (Shenmai injection plus chemotherapy) could significantly increase the proportion of CD^3+^ and CD^4+^ in the T-lymphocytes of patients with lung cancer. This suggested that the combined application of Shenmai injection and chemotherapy could enhance immune function in patients with lung cancer.

However, the current meta-analysis had some limitations. (1) The RCTs included in the present study lack follow-up visits owing to problems in the experimental design, leading to a lack of results concerning long-term efficacy, such as progression-free survival time, median survival time, and five-year survival rate. Consequently, the significance of the current meta-analysis is limited. (2) Multicentered, large-sized RCTs were not included in the present meta-analysis; the majority of the RCTs were small-scale trials. Consequently, clinical heterogeneity could not be avoided, and this may have affected the strength of the results and conclusions. (3) The experimental design was not sufficiently precise. Only seven studies described the randomization method that was used; this was not mentioned in other studies. None of the RCTs mentioned the blinding method or allocation concealment; thus, there may have been implementation bias and measurement bias. (4) All the included studies were conducted in China. Consequently, there was racial bias, which may also have affected the results of analysis. (5) The chemotherapies included in these RCTs were not consistent: even when the same treatment strategies were adopted, the treatment time and dosage were different, which may also have affected the results somewhat. (6) Some results in the present study were very highly heterogeneous, which may have affected the accuracy of the results.

## 5. Conclusion

The combination of Shenmai injection plus chemotherapy for lung cancer significantly increased the short-term efficacy of chemotherapy, improved patient quality of life, reduced toxic and adverse effects of chemotherapy, and enhanced the immune function of patients with lung cancer. Although the present meta-analysis included many studies and contained a large sample size in total, the results were convincing and the arguments were powerful, and there were several limitations. More multicenter, high-quality RCTs with larger sample sizes are required to confirm the results.

## Figures and Tables

**Table 1:** Characteristics of included articles.

Study	N (T/C)	Physical	Clinical stage	Treatment of experimental group	Treatment of control group	Outcomes
Cheng 2019 [12]	43/43	NR	III, IV	GP + SMI (50 mL/d, d1-d14)	GP	①③④⑤⑥⑦⑧⑨
Chen 2018 [13]	48/48	KPS ≥60	III, IV	DP + SMI (100 mL/d, d1-d21)	DP	①
Zhang 2018 [14]	30/30	NR	IIb, IIIa, IIIb	GP + SMI (100 mL/d, d1-d14）	GP	①②
Zhang 2018 [15]	57/57	KPS ≥60	I, II, III, IV	GP + SMI (50 mL/d, d1-d28)	GP	④⑤⑥
Xue 2007 [16]	30/30	KPS ≥60	III, IV	NP + SMI (60 mL/d, d1-d10)	NP	①
Zhou 2007 [17]	32/30	NR	IIIb, IV	GP + SMI (60 mL/d, d1-d21)	GP	①②④⑤⑥⑦⑧⑨
Hu 2008 [18]	40/40	KPS <70	III, IV	NP + SMI (40 mL/d, d1-d28)	NP	①③
Wei 2008 [19]	30/30	KPS ≥60	III, IV	NP + SMI (60 mL/d, d1-d14)	NP	①
Zhao 2008 [20]	56/56	KPS ≥60	NR	NP + SMI (50 mL/d, d1-d15)	NP	②④⑨
Feng 2009 [21]	85/80	NR	III, IV	GP + SMI (100 mL/d, d1-d15)	GP	②④⑥⑨
Shi 2008 [22]	30/30	KPS ≥60	III, IV	NP + SMI (60 mL/d, d1-d30)	NP	④⑤⑥⑨
Tong 2009 [23]	61/60	KPS ≥60	III, IV	GP + SMI OR TP + SMI OR NP + SMI(30 mL/d, d1-d10)	GP OR TP OR NP	①②④
Zhong 2009 [24]	31/30	KPS ≥50	III, IV	GP + SMI (50 mL/d, d1-d10)	GP	①②③
Guo 2010 [25]	31/31	KPS ≥70	IIIa, IIIb, IV	TP + SMI (50 mL/d, d1-d14)	TP	①②⑥⑨
Wei 2010 [26]	30/30	KPS ≥60	IIIa, IIIb, IV	TP + SMI (60 mL/d, d1-d30)	TP	②④⑥⑨
Zheng 2010 [27]	30/30	KPS ≥60	IIIb, IV	DP + SMI (60 mL/d, d1-d15)	DP	①④⑨
Chen 2011 [28]	64/50	KPS ≥60	IIIb, IV	GP + SMI OR TC + SMI OR NP + SMI (50 mL/d, d1-d14)	GP OR TC OR NP	⑤⑥
Ji 2011 [29]	20/20	NR	IIIb, IV	GP + SMI (60 mL/d, d1-d28)	GP	①④⑥⑨
Liu 2011 [30]	30/30	KPS ≥60	IIIb, IV	TP + SMI (60 mL/d, d1-d15)	TP	①②④⑦⑨
Lu 2011 [31]	30/30	KPS ≥60	IIIa, IIIb, IV	NP + SMI (50 mL/d, d1-d14)	NP	②④⑥
Fu 2012 [32]	30/30	KPS ≥60	IIIa, IIIb, IV	GP + SMI (60 mL/d, d1-d15)	GP	①④⑤⑥⑦⑧⑨
Li 2012 [33]	25/25	KPS ≥60	IIIb, IV	GP + SMI (50 mL/d, d1-d10)	GP	①④⑤⑦⑧⑨
Li 2013 [34]	54/53	KPS ≥60	IIIb, IV	GP + SMI (50 mL/d, d1-d10)	GP	①②④⑨
Shang 2012 [35]	25/25	KPS ≥60	IIIb, IV	GP + SMI (50 mL/d, d1-d10)	GP	①
Chen 2013 [36]	29/28	NR	IIIb, IV	TP + SMI (d1-d7)	TP	④⑥
Jiang 2013 [37]	30/30	KPS ≥60	IIIb, IV	GP + SMI OR DP + SMI (50 mL/d, d1-d14)	GP OR DP	②④⑥⑦⑧⑨
Yao 2013 [38]	24/24	KPS ≥60	IIIa, IIIb, IV	NP + SMI (40–60 mL/d, d1-d10)	NP + SMI	①
Zhang 2013 [39]	27/27	KPS ≥60	III, IV	NP + SMI (60 mL/d, d1-d14)	NP + SMI	④⑥
Shen 2014 [40]	25/25	KPS ≥60	IIIb, IV	DP + SMI (100 mL/d, d1-d14)	DP	①④⑤⑥
Yao 2014 [41]	40/40	NR	IIIb, IV	NP + SMI (60 mL/d, d1-d10)	NP	①④⑥⑨
Sun 2015 [42]	40/40	KPS ≥60	IIIb, IV	GP + SMI (40 mL/d, d1-d10)	GP	①②④⑤⑥
Hua 2016 [43]	40/40	NR	NR	GP + SMI (40 mL/d, d1-d7)	GP	①④⑥⑨
Li 2016 [44]	73/40	KPS ≥60	IIIa, IIIb, IV	GP + SMI (50 mL/d, d1-d10)	GP	①③
Wang 2016 [45]	24/24	KPS ≥60	IIIb, IV	TP + SMI (100 mL/d, d1-d7)	TP	①③④⑤
Xu 2016 [46]	23/23	KPS ≥60	IIIb, IV	GP + SMI (40 mL/d, d1-d10)	GP	①②
Luo 2017 [47]	81/81	NR	IIIa, IIIb, IV	DP + SMI (40 mL/d, d1-d30)	DP	③④⑥⑨
Wu 2017 [48]	30/30	KPS ≥70	III, IV	NP + SMI OR GP + SMI (50 mL/d, d1-d28)	NP OR GP	①④⑤⑥⑦⑧⑨

## Data Availability

The data used to support the findings of this study are available from the corresponding author upon request.
